# Risk factors for admission to the pediatric critical care unit among children hospitalized with COVID-19 in France

**DOI:** 10.3389/fped.2022.975826

**Published:** 2022-09-07

**Authors:** Blandine Prévost, Aurélia Retbi, Florence Binder-Foucard, Aurélie Borde, Amélie Bruandet, Harriet Corvol, Véronique Gilleron, Maggie Le Bourhis-Zaimi, Xavier Lenne, Joris Muller, Eric Ouattara, Fabienne Séguret, Pierre Tran Ba Loc, Sophie Tezenas du Montcel

**Affiliations:** ^1^Department of Pediatric Pulmonology, APHP Hôpital Trousseau, Sorbonne Université, Paris, France; ^2^Sorbonne Université, Centre de Recherche Saint Antoine (CRSA), Inserm UMR_S938, Paris, France; ^3^Department of Medical Information, Assistance Publique-Hôpitaux de Paris, Groupe Hospitalier Sorbonne Université, Paris, France; ^4^Department of Public Health, Strasbourg University Hospital, Strasbourg, France; ^5^Department of Medical Information, Medical Information Analysis and Coordination Unit (UCAIM - DIM), University Hospital Center Bordeaux, Bordeaux, France; ^6^Department of Medical Information, Lille University Hospital Center, Lille, France; ^7^Inserm U1219/Bordeaux Population Health Research Center, Population Health trAnslational Research (PHARes), University of Bordeaux, Bordeaux, France; ^8^Department of Medical Information, Hospices Civils de Lyon, Lyon, France; ^9^Unit of Evaluation and Epidemiologic Studies on National Hospitalization Databases, Department of Epidemiology, Biostatistics and Medical Information, University Hospital Center Montpellier, Montpellier, France; ^10^Department of Medical Information, Sorbonne Université, Institut du Cerveau—Paris Brain Institute—ICM, CNRS, Inria, Inserm, Assistance Publique-Hôpitaux de Paris, Groupe Hospitalier Sorbonne Université, Paris, France

**Keywords:** COVID-19, SARS-CoV-2, children, critical care, hospitalization

## Abstract

**Background:**

COVID-19 infection is less severe among children than among adults; however, some patients require hospitalization and even critical care. Using data from the French national medico-administrative database, we estimated the risk factors for critical care unit (CCU) admissions among pediatric COVID-19 hospitalizations, the number and characteristics of the cases during the successive waves from January 2020 to August 2021 and described death cases.

**Methods:**

We included all children (age < 18) hospitalized with COVID-19 between January 1st, 2020, and August 31st, 2021. Follow-up was until September 30th, 2021 (discharge or death). Contiguous hospital stays were gathered in “care sequences.” Four epidemic waves were considered (cut off dates: August 11th 2020, January 1st 2021, and July 4th 2021). We excluded asymptomatic COVID-19 cases, post-COVID-19 diseases, and 1-day-long sequences (except death cases). Risk factors for CCU admission were assessed with a univariable and a multivariable logistic regression model in the entire sample and stratified by age, whether younger than 2.

**Results:**

We included 7,485 patients, of whom 1988 (26.6%) were admitted to the CCU. Risk factors for admission to the CCU were being younger than 7 days [OR: 3.71 95% CI (2.56–5.39)], being between 2 and 9 years old [1.19 (1.00–1.41)], pediatric multisystem inflammatory syndrome (PIMS) [7.17 (5.97–8.6)] and respiratory forms [1.26 (1.12–1.41)], and having at least one underlying condition [2.66 (2.36–3.01)]. Among hospitalized children younger than 2 years old, prematurity was a risk factor for CCU admission [1.89 (1.47–2.43)]. The CCU admission rate gradually decreased over the waves (from 31.0 to 17.8%). There were 32 (0.4%) deaths, of which the median age was 6 years (IQR: 177 days–15.5 years).

**Conclusion:**

Some children need to be more particularly protected from a severe evolution: newborns younger than 7 days old, children aged from 2 to 13 years who are more at risk of PIMS forms and patients with at least one underlying medical condition.

## Introduction

Two years after the first cases, the severe acute respiratory syndrome coronavirus 2 (SARS-CoV-2) pandemic continues to spread around the world with the regular emergence of new variants of concern. Rapid dissemination of the delta variant (B.1.617.2) in the summer of 2021 and then of the omicron variant (B.1.1.529) in the winter of 2021–2022 were associated with an increase in children hospitalizations, which worries the pediatric community in several affected countries (1–4). Although this surge of pediatric hospitalizations seemed to be mainly attributable to higher contagiousness rather than to higher severity concerning the delta variant (5), the follow-up is ongoing for the omicron variant.

Even if morbidity has been much lower for children than for adults, the pediatric population has not been completely spared (6–14). Children could also be severely affected, requiring hospitalizations and intensive care unit admission, although deaths rarely occur in children (15). Several risk factors associated with these severe forms have already been identified, notably including young age and underlying medical conditions (6, 7, 13–16). Their precise understanding is important in the long-term management of the pandemic, both to identify children who should most benefit from adapted care and surveillance and to design and implement relevant public health policies, insofar as the psychological health of children is affected by these measures (17).

Some studies have already looked at pediatric hospitalizations and risk factors for severity, but none has provided a review of hospitalizations nationwide over a long period and taken into account contiguous hospital stays. Thus, the aim of our national observational study were since the outbreak of COVID-19 (from January 1st, 2020, to September 30th, 2021) to determine the risk factors associated with pediatric critical care unit (CCU) admissions. The secondary aims were to describe the number and characteristics of pediatric hospitalizations in all French hospitals and the clinical characteristics of children who died of COVID-19.

## Materials and methods

### Study design

We performed a retrospective nested case-control cohort analysis using data from the French national “*Programme de Médicalisation des Systèmes d’Information”* (PMSI) database (18). The PMSI is a comprehensive national database that gathers pseudonymized hospitalization data transmitted monthly by all public and private hospitals in France. Diagnoses are coded using the International Classification of Diseases, 10th Revision (ICD-10).

This study was conducted in accordance with French legislation concerning the reuse of the PMSI database (MR-005 of the Commission nationale de l’informatique et des libertés, CNIL), with an inscription on the Health Data Hub public register (N° F20201117130456). Patients were not involved, as we used pseudonymized discharge data.

We included data from all children (age < 18) admitted to French hospitals with COVID-19 between January 1st, 2020, and August 31st, 2021. Patients were followed up until discharge or death until September 30th, 2021. Hospital stays for COVID-19 were identified according to national guidelines (19; Supplementary Table 1). Acute COVID-19 cases were classified into three categories: respiratory form (ICD codes U07.10, U07.11), pediatric inflammatory multisystem syndrome (PIMS) (U10.9 or COVID-19 code associated with M30.3 (mucocutaneous lymph node syndrome [Kawasaki])) and “neither PIMS nor respiratory form” (U07.14, U07.15). If the case matched with many categories, the prioritization order was PIMS > respiratory form > neither PIMS nor respiratory form. We did not include asymptomatic COVID-19 cases and post-COVID-19 diseases (Supplementary Table 1). All contiguous hospital stays between hospitals for the same patient (<1 day between stays) were gathered and considered a unique “care sequence.” The care sequence starting date was the starting date of the first stay, and the ending date was the date of discharge of the last stay. Only the first sequence per patient was considered. In the context of COVID-19, France has adopted a massive screening strategy and these involved often scheduled stays with systematic screening outside the scope of our study. Consequently, we excluded care sequences lasting less than a day, except in cases of death.

### Outcomes

The primary outcome was the requirement for hospitalization in CCUs. The CCUs included intensive care units, intermediate care units (so-called “*soins intensifs*”), and step-down units (so-called “*unité de surveillance continue*”). The secondary outcome was in-hospital death.

### Covariates

The variables extracted for each patient were age, sex, dates of hospital admission and discharge, length of stay, chronic conditions, and admission to a CCU. The use of invasive or non-invasive ventilation, extracorporeal membrane oxygenation (ECMO), and vasoactive drugs were defined based on the act coded using the Common French Classification of Medical Acts (Supplementary Table 2).

The ICD-10 codes used to specify underlying chronic conditions were defined by a team of physicians experienced in medical information and a pediatrician and are listed in Supplementary Table 3. These chronic conditions were retrieved from the current care sequence and from the hospitalizations within the two previous years. The following 12 chronic conditions were considered: asthma, chronic lung disease excluding asthma, diabetes, metabolic disease excluding diabetes, sickle cell disease, obesity, cardiovascular disease, neurologic diseases, immunocompromised conditions including cancer, Down syndrome, hepatic and gastric disease, and renal disease. In addition, for children younger than 2 years old, prematurity was considered when one hospitalization within the previous 2 years contained one of the following ICD10 codes (P07.2, P07.3) or when the birth term was lower than 37 amenorrhea weeks.

### Statistical analysis

For continuous variables, data were described by their median and interquartile ranges [IQRs]. Categorical variables were described as numbers of patients and percentages. Age was also divided into age groups: 0–7 days, 8–89 days, 90 days-1 year, 2–9 years, 10–13 years, and 14–17 years. For global chronological description, the time unit used was the month of the sequence’s starting date. Four periods, corresponding to 4 different epidemic waves, were considered from January 1st to August 11th 2020, from August 12th 2020 to January 1st 2021, from January 2nd to July 4th 2021 and from July 5th to August 31st 2021.

Qualitative variables were compared between groups using Chi-square tests, and quantitative variables were compared using ANOVA. Risk factors for CCU admission were assessed with a univariable and a multivariable logistic regression model in the entire sample and stratified by age, whether younger than 2. Multivariable models included all the variables with at least 10 patients per modality.

The analyses were performed on the secure platform of the ATIH. Data extraction and preparation were carried out on January 11th 2022 with SAS Enterprise Guide version 8.3. *P*-values lower than 0.05 were considered statistically significant.

## Results

### Study population

**FIGURE 1:**
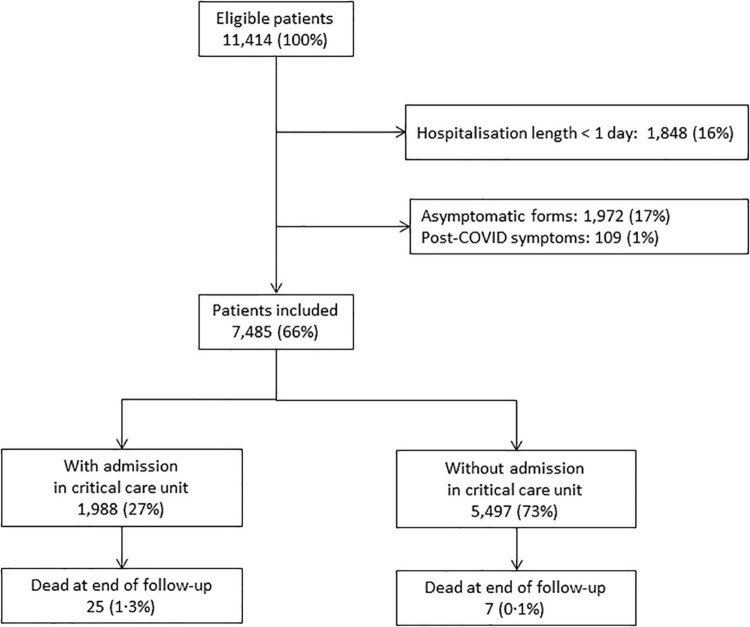
Study patient selection flow chart.

We identified 11,414 patients younger than 18 years who were hospitalized with SARS-CoV-2 infection in France between March 2020 and August 2021. We excluded 1,848 (16.2%) patients who were discharged alive after a length of stay less than 1 day, 1,972 (17.3%) patients with asymptomatic infections and 109 (1.0%) hospitalizations related to post-COVID symptoms. The characteristics of the excluded patients are given in Supplementary Table 4. They were more frequently younger than 6 days and older than 10 years old than the included patients. In addition, they had less comorbidities, except for diabetes that was twice more frequent. Finally, 7,485 children were included, all of whom had been admitted to a hospital with a symptomatic SARS-CoV-2 infection. The median duration of hospitalization was 3 days (IQR 1–5); 1,988 (26.6%) were admitted to CCUs, and 32 (0.4%) died (Figure 1).

### Demographic and clinical characteristics

**FIGURE 2:**
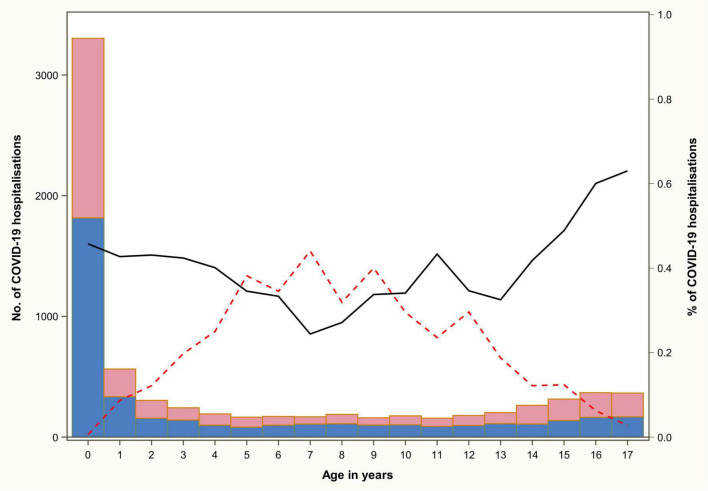
Number of COVID-19 hospitalizations and of the PIMS and respiratory forms according to the age and gender of the patient. The bars give the number of COVID-19 hospitalizations for the males (blue) and the females (pink) separately. The lines give the proportion of the PIMS (red dotted line) and respiratory (black plain line) forms among the hospitalizations.

The median age of symptomatic hospitalized children was 1.0 year (IQR: 57 days-10 years); 32.0% were under 3 months old (*n* = 2,392) (Table 1). Most children were male (53.8%) (Figure 2 and Supplementary Figure 1). No underlying medical condition was identified in 71.9% of hospitalized children, even though this proportion varied in the different age groups to be minimal for the 2–9-year-old group (61.2%). For all these children, the most common underlying condition was chronic lung disease, including asthma (9.0%), followed by immunocompromised conditions (4.9%), neurologic diseases (4.8%) and metabolic disease (4.7%). Other underlying conditions appear in Table 1. Prematurity was reported for 375 children younger than 2 years old, i.e., 9.7% of this age group.

**TABLE 1:** Characteristics of the children hospitalized with COVID-19.

	All Children (*n* = 7,485)	With CCU (*n* = 1,988)	Without CCU (*n* = 5,497)
Male gender	4,025 (53.8%)	1,115 (56.1%)	2,910 (52.9%)
Age, median (IQR)	1 Year (57 days–10 years)	4 Years (105 days–11 years)	1 Year (53 days–10 years)
**Age groups**			
0–6 days	130 (1.7%)	68 (3.4%)	62 (1.1%)
7 days to less than 3 months	2,262 (30.2%)	412 (20.7%)	1,850 (33.6%)
3 months to less than 2 years	1,476 (19.7%)	312 (15.7%)	1,164 (21.2%)
2–9 years	1,591 (21.3%)	609 (30.6%)	982 (17.9%)
10–13 years	715 (9.6%)	255 (12.8%)	460 (8.4%)
14–17 years	1,311 (17.5%)	332 (16.7%)	979 (17.8%)
**Underlying medical condition**			
Respiratory disease	671 (9.0%)	254 (12.8%)	375 (6.8%)
Asthma	468 (6.3%)	186 (9.4%)	282 (5.1%)
Chronic lung disease excluding asthma	203 (2.7%)	86 (4.3%)	117 (2.1%)
Metabolic disease	353 (4.7%)	163 (8.2%)	176 (3.2%)
Diabetes	96 (1.3%)	29 (1.5%)	67 (1.2%)
Metabolic disease excluding diabetes	257 (3.4%)	140 (7.0%)	117 (2.1%)
Sickle-cell disease	211 (2.8%)	90 (4.5%)	121 (2.2%)
Obesity	107 (1.4%)	42 (2.1%)	65 (1.18%)
Cardiovascular disease	275 (3.7%)	143 (7.2%)	132 (2.4%)
Neurologic disease	361 (4.8%)	147 (7.4%)	214 (3.9%)
Immunocompromised condition (including cancers)	363 (4.9%)	129 (6.5%)	234 (4.3%)
Hepatic and gastric disease	48 (0.6%)	14 (0.7%)	34 (0.6%)
Renal disease	45 (0.6%)	15 (0.8%)	30 (0.5%)
Down syndrome	42 (0.6%)	15 (0.8%)	27 (0.5%)
Prematurity among < 2 years (*n* = 3,868)	375 (9.7%)	129 (16.3%)	246 (8.0%)
**Number of underlying conditions**			
0	5,380 (71.9%)	1,145 (57.6%)	4,235 (77.0%)
1	1,597 (21.3%)	621 (31.2%)	976 (17.8%)
2	367 (4.9%)	162 (8.1%)	205 (3.7%)
3 or more	141 (1.9%)	60 (3.0%)	81 (1.5%)
**COVID-19 forms**			
PIMS	806 (10.8%)	512 (25.8%)	297 (5.4%)
Respiratory form	3,294 (44.0%)	838 (42.2%)	2,456 (44.7%)
No PIMS no respiratory form	3,385 (45.2%)	638 (32.1%)	2,747 (50.0%)
**Level of care required**			
Admission in critical care unit	1,988 (26.6%)		
Ventilation	461 (6.2%)	387 (19.5%)	74 (1.3%)
Invasive ventilation	149 (2.0%)	133 (6.7%)	16 (0.3%)
Non-invasive ventilation	385 (5.1%)	320 (16.1%)	65 (1.2%)
ECMO	7 (0.1%)	7 (0.4%)	0 (0.0%)
Need of vasoactive drugs	241 (3.2%)	204 (10.3%)	37 (0.7%)
Hospital length of stay, median (IQR), days	3 (1–5)	6 (3–10)	2 (1–4)

IQR, interquartile range; CCU, critical care unit; ECMO, extracorporeal membrane oxygenation; PIMS, pediatric multisystem inflammatory syndrome.

### COVID-19 forms

Respiratory forms represented 44.0% of hospitalizations (*n* = 3,294). The PIMS rate was 10.8% (*n* = 806) for all admitted children, with a maximum rate of 44.0% for the 7-year-old children (Figure 2). In fact, the 2–9-year-old group accounted for 56.2% (*n* = 453) of all PIMS. The proportion of PIMS and respiratory forms of COVID-19 hospitalized by age is shown in Figure 2. The demographic and clinical characteristics of the different forms of COVID-19 are presented in Supplementary Table 5.

### Risk factors for critical care unit admission

**FIGURE 3:**
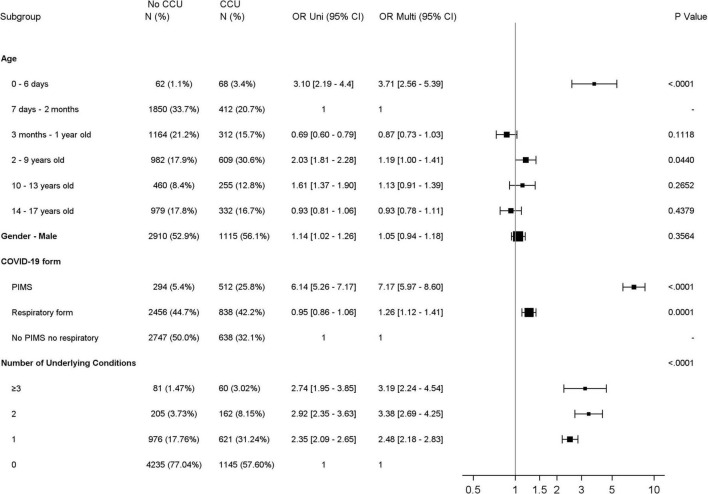
Forest plot showing the odds ratio using Univariable (Uni) and Multivariable (Multi) Logistic regression analysis for CCU admission for all children. CCU, critical care unit; PIMS, pediatric multisystem inflammatory syndrome; OR, Odds ratio; 95% CI, 95% confidence interval.

Over the entire analysis period, 461 (6.2%) children required ventilatory support (Table 1). Seven children were treated by ECMO. The use of vasoactive drugs was reported for 241 patients (3.2%), among whom 171 (71.0%) had PIMS. Risk factors for admission to CCUs evidenced by multivariate analyses (Figure 3 and Supplementary Table 6) included age younger than 7 days old [OR: 3.71 95% CI (2.56–5.39)] and age between 2 and 9 years old [1.19 (1.00–1.41)]; and PIMS [7.17 (5.97–8.6)] and respiratory forms [1.26 (1.12–1.41)] compared to “neither PIMS nor respiratory form.” The presence of at least one underlying condition increased the risk of CCU admission [2.66 (2.36–3.01)]. Sex was not associated with CCU admission [1.05 (0.94–1.18)].

**FIGURE 4:**
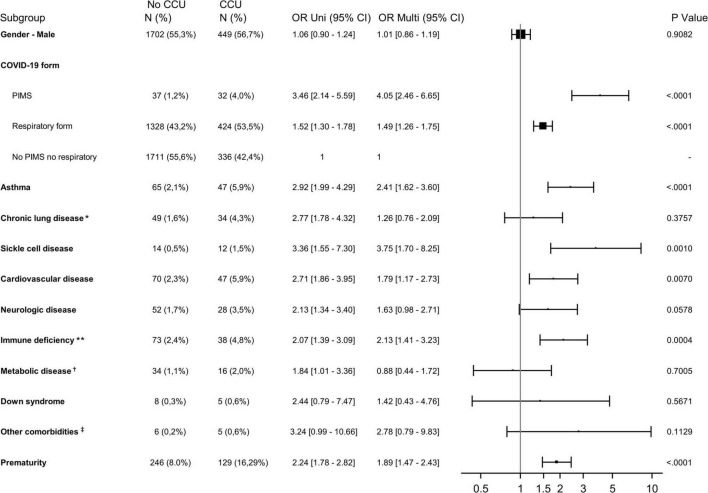
Forest plot showing the odds ratio using univariable (Uni) and multivariable (Multi) logistic regression analysis for CCU admission for children younger than 2 years old. CCU, critical care unit; PIMS, pediatric multisystem inflammatory syndrome; OR, odds ratio; 95% CI, 95% confidence interval. *Excluding asthma; **including cancers; ^†^excluding diabetes; ^‡^renal disease, hepatic and gastric disease, and diabetes.

As prematurity was assessed only for children younger than 2 years old, the analysis of risk factors for CCU admission was stratified according to the age of children (<> 2 years old). Among hospitalized children younger than 2 years old, most underlying conditions identified were significantly associated with CCU admissions except for Down syndrome, chronic lung disease excluding asthma, and neurologic and metabolic disease (Figure 4 and Supplementary Table 7). Prematurity, the most common underlying condition in this age group (16.3% of those admitted to the CCU), was identified as a risk factor for CCU admission [1.89 (1.47–2.43)].

**FIGURE 5:**
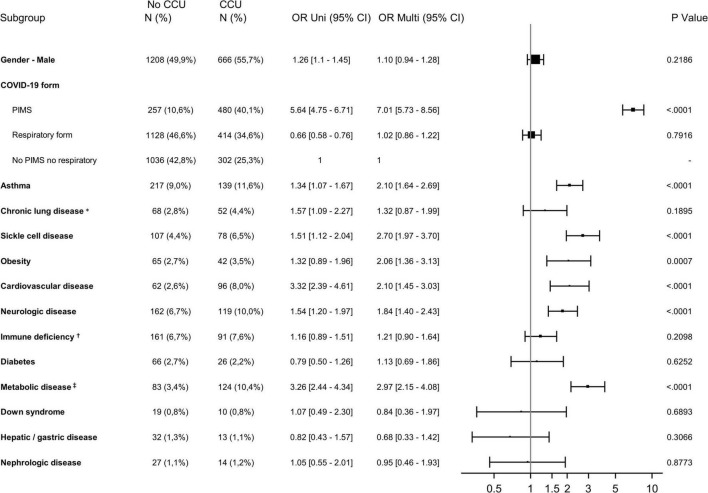
Forest plot showing the odds ratio using univariable (Uni) and multivariable (Multi) logistic regression analysis for CCU admission for children older than 2 years old. CCU, critical care unit; PIMS, pediatric multisystem inflammatory syndrome; OR, odds ratio; 95% CI, 95% confidence interval. *Excluding asthma; ^†^including cancers; ^‡^excluding diabetes.

Among those older than 2 years old, metabolic disease [2.97 (2.15–4.08)], sickle cell disease [2.70 (1.97–3.70)], asthma [2.10 (1.64–2.69)], cardiovascular disease [2.10 (1.45–3.03)], obesity [2.06 (1.36–3.13)] and neurologic disease [1.84 (1.40–2.43)] significantly increased the risk of CCU admission (Figure 5 and Supplementary Table 7). In this group, Down syndrome and chronic lung disease excluding asthma were not found to be at risk either.

### Pediatric deaths

Among the children in the study, 32 (0.4%) died during their hospital stay (Table 2). The median age of the deceased patients was 6.0 years (IQR: 177 days–15.5 years). Four had no underlying condition identified, and among the 28 others (87.5%), neurologic disorders were the most common (*n* = 19, 59.4%), followed by chronic lung disease (*n* = 10 including 4 with asthma, 31.3%), metabolic disease (*n* = 7, 21.9%) and immune deficiency (*n* = 6, 18.8%). Five of those who died had a cardiovascular disease (15.6%), and one was obese (3.1%). None had Down syndrome, sickle cell disease, or a chronic renal or digestive disease. Among the children younger than 2 who died (*n* = 13), 5 were premature. Regarding the distribution over time, 14 deaths occurred during the first wave (until August 2020), 5 during the second wave, 7 during the third wave and 6 during the fourth wave.

**TABLE 2:** Characteristics of the deceased children hospitalized with COVID-19.

	Deceased (*n* = 32)
Male gender	16 (50.0%)
Age, median (IQR)	6 Years (177 days–15.5 years)
**Age groups**	
0–6 days	4 (12.5%)
7 days to less than 3 months	1 (3.1%)
3 months to less than 2 years	8 (25.0%)
2–9 years	5 (15.6%)
10–13 years	2 (6.3%)
14–17 years	12 (37.5%)
**Underlying medical condition**	
Respiratory disease	10 (31.3%)
Asthma	4 (12.5%)
Chronic lung disease excluding asthma	6 (18.8%)
Metabolic disease	7 (21.9%)
Diabetes	0
Metabolic disease excluding diabetes	7 (21.9%)
Sickle-cell disease	0
Obesity	1 (3.1%)
Cardiovascular disease	5 (15.6%)
Neurologic disease	19 (59.4%)
Immunocompromised condition (including cancers)	6 (18.8%)
Hepatic and gastric disease	0
Renal disease	0
Down syndrome	0
Prematurity among < 2 years (*n* = 3868)	5 (38.5%)
**Number of underlying conditions**	
0	4 (12.5%)
1	13 (40.6%)
2	8 (25.0%)
3 Or more	7 (21.9%)
**COVID-19 forms**	
PIMS	1 (3.1%)
Respiratory form	25 (78.1%)
No PIMS no respiratory form	6 (18.8%)
**Level of care required**	
Admission in critical care unit	25 (78.1%)
Ventilation	23 (71.9%)
Invasive ventilation	20 (62.5%)
Non-invasive ventilation	9 (28.1%)
ECMO	4 (12.5%)
Need of vasoactive drugs	12 (37.5%)
Hospital length of stay, median (IQR), days	8 (2–20.5)

IQR, interquartile range; CCU, critical care unit; ECMO, extracorporeal membrane oxygenation; PIMS, pediatric multisystem inflammatory syndrome.

### Hospitalizations over time (waves)

**FIGURE 6:**
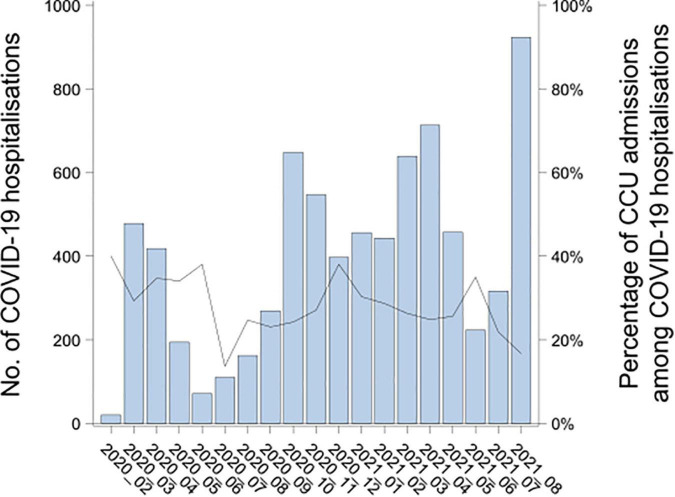
Number of pediatric COVID-19 hospitalizations by month and percentage of COVID-19 hospitalizations in critical care units in France from February 2020 to August 2021. The bars give the number of COVID-19 hospitalizations per month. The black plain line gives the percentage of CCU admissions among the COVID-19 hospitalizations.

Symptomatic COVID-19 admissions among children in French hospitals peaked in March 2020 (first wave), October 2020 (second wave), April 2021 (third wave) and August 2021 (fourth wave), with 478, 648, 713, and 923 monthly admissions, respectively (Figure 6). The percentage of hospitalizations with admission to CCUs among all hospitalizations gradually decreased over the different waves: Starting with 31.0% admitted to CCU, then 27.6, 27.5, and 17.8%, respectively. The mean age decreased from 6.0 ± 0.2 years old during the first wave to 3.8 ± 0.2 years old during the fourth wave (*p* < 0.001).

## Discussion

The national PMSI database enabled the exhaustive analysis of 7,485 pediatric hospitalizations for symptomatic SARS-CoV-2 infection from January 2020 to September 2021 in France. This study, gathering the largest number of symptomatic children hospitalized over the longest period ever assessed, strengthens knowledge of pediatric COVID-19 and highlights risk factors for severe disease. More than one-fourth of these children required admission to the CCU, and risk factors identified by multivariate analyses were (i) being younger than 7 days old or from 2 to 9 years old and (ii) PIMS or “respiratory form.” Most underlying conditions were significantly associated with CCU admissions. Unlike adults, Down syndrome and diabetes were not significantly associated (20, 21).

The rate of admissions to the CCU (26.6%) was in the upper range of those reported in similar pediatric studies between 4.1 and 30.1% (5, 7, 13, 22). This high rate may be partly explained by the methodology of this study and the choice to exclude asymptomatic hospitalized COVID-19 children, who were considered incidental findings. Another explanatory factor relates to the organization of care in France at the beginning of the pandemic, when all children infected by SARS-CoV-2 and requiring hospitalizations were grouped together in dedicated wards within the CCU. This is supported by the highest rate of hospitalizations in the CCU during the first wave in France (31%), when the traditional care organization was redeployed. Overall, this decrease in the CCU rate over time is another reassuring element in the analysis of the severity of the various variants of concern.

The analysis of CCU admission revealed an increased risk for patients younger than 7 days old. The fragility of this age group is presumably partly linked to particular neonatal contexts (prematurity, fetal distress, etc.) (23). Indeed, 37% of the hospitalized infants younger than 7 days old were premature. The involvement of SARS-CoV-2 infection in the vulnerability of these new-borns is not certain but should nevertheless attract our attention when it also appears that neonates without any history or underlying condition can present severe forms such as pneumonia (23, 24). This fragility was not confirmed in our analysis for infants older than 7 days old, while children younger than 2 years old represented more than half of the hospitalizations in this study. The follow-up of more than a year and a half in our study invalidated the first worrisome data considering infants as a vulnerable group (9). Regarding the 2–9-year-old and 10–13-year-old groups, we found higher odds of admission to the CCU in the univariate analysis, similar to another study (25). However, this was not confirmed by multivariate analyses, and it appeared that these age groups were more often affected by PIMS and therefore more at risk of requiring CCU without having a real over-risk (16, 22, 26).

The analysis of underlying respiratory conditions did not reveal any excess risk of CCU associated with chronic respiratory diseases other than asthma, and the involvement of these comorbidities in severe forms of infection is not yet clearly documented for all of them (7). Among patients with chronic respiratory diseases, those with cystic fibrosis were observed in several studies, and the increased risk associated with this pathology mainly concerned the most severe or the posttransplant patients (27). Moreover, these stages of disease concern more adults than children. Asthma was identified here as a risk factor for CCU admission for both age groups (<> 2 years old). Recent studies have also shown the involvement of asthma in the risk of hospital admission but not of CCU admission (7, 28). This increased risk of hospitalization, primarily for children with poorly controlled asthma (29), was not related to asthma severity, and SARS-CoV-2 infection did not appear to be a trigger exacerbation of asthma (28). Our study also gathered 211 children with sickle cell disease. France is the country with the highest prevalence of sickle cell disease in Europe, and we were able to highlight that this disease was associated with a significantly increased risk of CCU admission for children (30). Clinical courses were favorable for all patients. More generally, we have demonstrated a significant excess risk of CCU for children with at least one underlying medical condition. The highlighted underlying conditions can vary among the different previous studies, depending on the proportions of each pathology in the different countries and the differences in underlying condition definitions in the studies. It should be remembered that a child followed for a chronic pathology is more at risk of presenting with severe COVID-19 (7, 13, 14, 16).

In pediatrics, the youngest children, including new-borns and those with comorbidities, are the most vulnerable to respiratory infections (31, 32), and SARS-CoV-2 infection is no exception to the rule. Those children are more at risk of presenting severe complications and of admission to the CCU. The unusual aspect of this pandemic in pediatrics is that healthy middle-aged children can develop serious complications of SARS-CoV-2 infection, such as PIMS, and be hospitalized to CCU. In addition, long COVID-19, which was not described in this study, also seems to affect healthy children older than 6 years old (33). Highlighting these pediatric specificities should encourage families and health professionals to vaccinate all children and pregnant women, above all those with underlying conditions. The safety and efficiency of the BNT162b2 COVID-19 vaccine is high, and very rare adverse effects were indeed observed (34). In France, convincing parents to provide COVID-19 vaccination for children aged 5–11 years old is a struggle.

A limitation of the PMSI national database is the possible variation in the quality of coding. We may be confident in the quality of the collected data because national COVID-19 coding guidelines have been published shortly after the beginning of the pandemic. Moreover, strict national rules with regular checks carried out by the payer may limit the risk of coding errors. The PMSI database does have many advantages, such as being exhaustive and national, including all the data from public and private hospitals. In addition, this study allowed us to gather continuous stays within different hospitals so that the risk of overestimating the number of hospitalizations is limited. We included all patients presenting any symptoms of COVID-19 regardless of the severity during their stay if an ICD-10 code was present in their records. If the patients became symptomatic after admission, then an ICD-10 code of symptomatic COVID-19 was present in the file and the patient was considered.

## Conclusion

In conclusion, the SARS-CoV-2 pandemic among children is not comparable to the adults’ in terms of hospitalization and mortality rates. Nevertheless, childhood morbidity is not negligible, as evidenced by the number of hospitalizations for symptomatic forms in France, the high rate of CCU admission and the number of deaths. We are able to confirm that some children are particularly at risk of evolving toward a severe infection: new-borns younger than 7 days old, children aged from 2 to 13 years who are more at risk of PIMS, and patients with at least one underlying medical condition. Repercussions on the child’s overall health are also a constant concern of pediatricians and should of course guide the long-term management of the pandemic.

## Data availability statement

Raw data are available upon request to the Agence technique de l’information sur l’hospitalisation (ATIH): support@atih.sante.fr. Aggregated data can be provided upon request to the authors.

## Ethics statement

Ethical review and approval was not required for the study on human participants in accordance with the local legislation and institutional requirements. Written informed consent from the participants’ legal guardian/next of kin was not required to participate in this study in accordance with the national legislation and the institutional requirements.

## Author contributions

BP, AR, FB-F, PT, and ST involved in the methodology, formal analysis, investigation, data curation, writing the original draft, reviewing and editing the manuscript, designing of tables and graphs, and verified the underlying data. ABo, ABr, HC, VG, ML, XL, JM, EO, and FS involved in data provision and reviewing and editing the manuscript. All authors had full access to all data in the study and accept responsibility for the decision to submit for publication.
