# Supplementary material for: Risk factors for admission to the pediatric critical care unit among children hospitalized with COVID-19 in France
Source: Front Pediatr. 2022 Sep 7;10:975826. doi: 10.3389/fped.2022.975826 (PMC9489832; doi:10.3389/fped.2022.975826)
Supplement: Supplementary file 1 [file Data_Sheet_1.docx]

Supplementary Material

### **Supplementary Table 1.** Codes of the International Classification of the Diseases (ICD), 10th revision, modified by the ATIH, used to identify the COVID cases in the PMSI database

### **Supplementary Table2.** Acts considered to describe the burden of the disease

### **Supplementary Table 3.** Codes of the International Classification of the Diseases (ICD), 10th revision, modified by the ATIH, used to identify children with chronic conditions

### **Supplementary Table 4.** Comparison of age distribution and comorbidities between the included and excluded patients

### **Supplementary Table 5.** Demographics and clinical characteristics according to the COVID-19 form

### **Supplementary Table 6**. Results from uni and multivariable regressions for CCU admission – Entire sample

### **Supplementary Table 7.** Results from uni and multivariable regressions for CCU admission – stratified by age either younger than 2 years old or not

**Supplementary Figure 1.** Number of COVID-19 hospitalizations and of the PIMS (red dotted line) and respiratory (black plain line) forms according to the age and gender of the patient younger than one-year-old

### **Supplementary Table 1.** Codes of the International Classification of the Diseases (ICD), 10th revision, modified by the ATIH, used to identify the COVID cases in the PMSI database

| **ICD 10 Code** | **Label** |
| --- | --- |
| U07.10 | COVID-19, respiratory form, confirmed case |
| U07.11 | COVID-19, respiratory form, unconfirmed case |
| U07.14 | COVID-19, other clinical form, confirmed case |
| U07.15 | COVID-19, other clinical form, unconfirmed case |
| U10.9 | Multisystem inflammatory syndrome associated with COVID-19, unspecified |

The following codes were not included in the definition of cases

| **ICD 10 Code** | **Label** |
| --- | --- |
| U07.12 | COVID-19, without symptoms, confirmed case |
| U09.9 | Post-COVID-19 disease, without precision |

### **Supplementary Table 2.** Acts considered to describe the burden of the disease

| **Label** | **Common French Classification of Medical Acts** |
| --- | --- |
| Use of invasive ventilation | GLLD004, GLLD015, GLLD008, GLLD009, GLLD007 |
| Use of noninvasive ventilation | ZZEP002, ZZEP003, ZZEP004, GLLD012, GLLD019, GLLD003, GLLD002, GLLD016, GLLD018 |
| Extracorporeal membrane oxygenation (ECMO) | EQLA002, GLJF010 |
| Vasoactive drugs | EQLF001, EQLF003 |

### **Supplementary Table 3. Codes of the International Classification of the Diseases (ICD), 10th revision, modified by the ATIH, used to identify children with chronic conditions**

| **Type of Chronic condition** | **Categories** | **Codes** |
| --- | --- | --- |
| Cancer/blood disorders | Sickle cell disease | D57.x |
|  | Immunocompromised condition (including cancer) | C00.x-C97.x, D46.6, D46.7, D46.9, D60.x*, D61.0, D61.1*, D61.2*, D61.3, D61.8*, D61.9*, D70*, D71, D76.1, D76.3, D80.x-D84.x, M05.2, M08.xx, M31.x-M35.x, M36.8, M45.xx, Q90.x, Z85.x, Z86.2, Z92.6, Z94.x, Z94.8x* |
| Respiratory disease | Asthma | J45.x, J46† |
|  | Chronic lung disease (excluding asthma) | E84.x, G47.3, J41.x-J44.x, J47, J60-J67.x, J70.3, J70.4, J82, J84.1-J84.9†, J95.3, J96.1x, J99.1, P27.1, P27.8, Q32.x, Q33.x, Q34.8, Q39.0-Q39.2, Q79.0, Z87.01 |
| Metabolic disease | Diabetes | E10.x, E11.x, E13.x, E14.x |
|  | Metabolic disease (excluding diabetes) | E03.0, E03.1, E03.8, E03.9, E05.0-E05.3, E05.8, E06.3, E16.8, E20.1, E20.8, E21.0-E21.3, E22.0, E22.1, E23.0, E24.x, E25.x, E27.0-E27.2, E28.xE31.0, E55.0, E70.1, E70.3, E71.x, E72.x, E74.x-E77.x, E78.0, E78.1, E78.5, E78.8, E79.0, E80.0, E80.4, E80.6, E83.0, E83.1, E83.3, E83.4, E85.x |
| Obesity | | E66.04-E66.09, E66.14-E66.19, E66.24-E66.29, E66.84-E66.89, E66.94-E66.99 |
| Cardiovascular disease | | I05.x-I09.x, I10, I11.x†, I13.x†, I20.x-I24.x†, I25.x, I27.1, I28.8, I34.0, I34.8, I35.0, I35.1, I36.1, I42.x†, I43.1, I44.x†, I45.x†, I47.x-I50.x†, I51.3†, I51.8†, I51.9†, Q20.x-Q23.x, Q24.5, Q24.8, Q24.9, Q25.1-Q25.9, Q26.0, Q26.2, Q26.3, Q26.4, Q26.8, Z86.71 |
| Neurologic disease | | F02.8x, F06.70, F06.71, F7x.x, F80.x-F84.x, F90.x, F92.9, G11.x-G13.x, G23.x, G24.1, G24.2, G25.0, G31.x, G32.x, G35, G36.0†, G37.3†, G37.8, G37.9, G40.0-G40.5, G40.8, G40.9, G60.x, G70.x-G72.x, G73.6, G80.x, G81.x-G83.x†, G90.4, G90.9, G91.x-G92.x, G93.1, G93.4, G94.3, G95.0, H49.4, I60.x-I64.x†, I69.8, P91.0, P91.6, Q03.x, Q04.0, Q04.3, Q04.4, Q04.8, Q04.9, Z86.5, Z86.61, Z86.70 |
| Down syndrome | | Q90.x |
| Hepatic and gastric disease | | B18.x, K50.x, K51.x, K72.1, K74.x, K75.4, K75.8, K86.1x, K90.0 |
| Renal disease | | N03.30, N04.x, N18.x |
| Prematurity | | P07.2, P07.3 or 0 to 36 amenorrhea weeks |

*on COVID-19 sequence only

†on last 2-year sequence excluding the COVID-19 sequence

### **Supplementary Table 4. Comparison of age distribution and comorbidities between the included and excluded patients**

|  | **Included Children (n=7525)** | **Excluded Children (n=3946)** | **p value** |
| --- | --- | --- | --- |
| Age groups |  |  | < 0.0001 |
| - 0 to 6 days | 131 (1.7%) | 120 (3.0%) |  |
| - 7 days to less than 3 months | 2282 (30.3%) | 375 (9.5%) |  |
| - 3 months to less than 2 years | 1479 (19.7%) | 416 (10.5%) |  |
| - 2 to 9 years | 1602 (21.3%) | 736 (18.7%) |  |
| - 10 to 13 years | 715 (9.5%) | 623 (15.8%) |  |
| - 14 to 17 years | 1316 (17.5%) | 1676 (42.5%) |  |
| Underlying medical condition: |  |  |  |
| - Respiratory disease | 631 (8.4%) | 177 (4.5%) | <0.0001 |
| - Asthma | 470 (6.3%) | 124 (3.1%) | <0.0001 |
| - Chronic lung disease excluding asthma | 203 (2.7%) | 65 (1.7%) | 0.0004 |
| - Metabolic disease | 341 (4.5%) | 153 (3.9%) | 0.1011 |
| - Diabetes | 96 (1.3%) | 84 (2.1%) | 0.0005 |
| - Metabolic disease excluding diabetes | 259 (3.4%) | 83 (2.1%) | <0.0001 |
| - Sickle-cell disease | 211 (2.8%) | 55 (1.4%) | <0.0001 |
| - Obesity | 108 (1.4%) | 36 (0.9%) | 0.0169 |
| - Cardiovascular disease | 275 (3.7%) | 76 (1.9%) | <0.0001 |
| - Neurologic disease | 365 (4.9%) | 196 (5.0%) | 0.7833 |
| - Immunocompromised condition (including cancers) | 368 (4.9%) | 126 (3.2%) | <0.0001 |
| - Hepatic and gastric disease | 49 (0.7%) | 28 (0.7%) | 0.7159 |
| - Renal disease | 45 (0.6%) | 19 (0.5%) | 0.4261 |
| - Down syndrome | 42 (0.6%) | 9 (0.2%) | 0.0116 |
| - Prematurity among < 2 years | 376 (5.0%) | 105 (2.7%) | <0.0001 |
| Number of underlying conditions |  |  | <0.0001 |
| 0 | 5409 (71.9%) | 3190 (80.8%) |  |
| 1 | 1603 (21.3%) | 590 (15.0%) |  |
| 2 | 372 (4.9%) | 119 (3.0%) |  |
| 3 or more | 141 (1.9%) | 47 (1.2%) |  |

Of notice, the sample size in this table changed little compared to the others data because we had to do a new data extraction from an update database. This has little impact on the results.

### **Supplementary Table 5. Demographics and clinical characteristics according to the COVID-19 form**

| **Patients characteristics** | **PIMS**  **(n=806)** | **Respiratory form**  **(n=3294**) | **No PIMS no respiratory form**  **(n=3385**) | **p value** |
| --- | --- | --- | --- | --- |
| Male gender | 493 (61.2%) | 1749 (53.1%) | 1783 (52. 7%) | <0.0001 |
| Deceased | 1 (0.1%) | 25 (0.8%) | 6 (0.2%) | 0.0005 |
| Age, mean ± SD | 7.9 ± 0.2 | 5.2 ± 0.1 | 4.1 ± 0.1 | <0.0001 |
| Age, median (IQR) | 8 [4-11] | 1 [0-12] | 0 [0-8] |  |
| Age groups |  |  |  | <0.0001 |
| 0 to 6 days old | 1 (0.1%) | 75 (2.3%) | 54 (1.6%) |  |
| 7 days to 2 months old | 3 (0.4%) | 980 (29.8%) | 1279 (37.8%) |  |
| 3 months to 1 year old | 65 (8.1%) | 697 (21.2%) | 714 (21.1%) |  |
| 2 to 9 years old | 453 (56.2%) | 571 (17.3%) | 567 (16.8%) |  |
| 10 to 13 years old | 180 (22.3%) | 256 (7.8%) | 279 (8.2%) |  |
| 14 to 17 years old | 104 (12.9%) | 715 (21.7%) | 492 (14.5%) |  |
| Level of care required |  |  |  |  |
| Admission in critical care unit (CCU) | 512 (63.5%) | 838 (25.4%) | 638 (18.9%) | <0.0001 |
| Ventilation | 93 (11.5%) | 286 (8.7%) | 82 (2.4%) | <0.0001 |
| Invasive ventilation | 37 (4.59%) | 78 (2.37%) | 34 (1.00%) | <0.0001 |
| Noninvasive ventilation | 74 (9.18%) | 253 (7.68%) | 58 (1.71%) | <0.0001 |
| ECMO | 0 (0.0%) | 5 (0.12%) | 2 (0.1%) | 0.3040 |
| Need of vasoactive drugs | 171 (21.2%) | 39 (1.2%) | 31 (0.92%) | <0.0001 |
| Hospital length of stay, median (IQR), days | 7 [5-10] | 3 [1-5] | 2 [1-4] | <0.0001 |
| Underlying medical condition |  |  |  |  |
| Asthma | 14 (1.7%) | 376 (11.4%) | 78 (2.3%) | <0.0001 |
| Chronic lung disease excluding asthma | 10 (1.2%) | 146 (4.4%) | 47 (1.4%) | <0.0001 |
| Diabetes | 4 (0.50%) | 34 (1.03%) | 58 (1.7%) | 0.0052 |
| Metabolic disease excluding diabetes | 62 (7.7%) | 97 (2.9%) | 98 (2.9%) | <0.0001 |
| Sickle-cell disease | 2 (0.3%) | 110 (3.3%) | 99 (2.9%) | <0.0001 |
| Obesity | 6 (0.7%) | 83 (2.5%) | 18 (0.5%) | <0.0001 |
| Cardiovascular disease | 73 (9.1%) | 120 (3.6%) | 82 (2.4%) | <0.0001 |
| Neurologic disease | 26 (3.2%) | 183 (5.6%) | 152 (4.5%) | 0.0103 |
| Immunocompromised condition | 30 (3.7%) | 148 (4.5%) | 185 (5.5%) | 0.0521 |
| Hepatic and gastric disease | 5 (0.6%) | 14 (0.4%) | 29 (0.9%) | 0.0868 |
| Renal disease | 4 (0.5%) | 15 (0.5%) | 26 (0.8%) | 0.2348 |
| Down syndrome | 1 (0.1%) | 30 (0.9%) | 11 (0.3%) | 0.0013 |
| Prematurity among < 2 years | 7 (10.1%) | 218 (12.4%) | 150 (7.3%) | <0.0001 |
| Number of underlying conditions |  |  |  | <0.0001 |
| 0 | 608 (75.4%) | 2173 (66.0%) | 2599 (76. 8%) |  |
| 1 | 164 (20.4%) | 824 (25.0%) | 609 (18.0%) |  |
| 2 | 28 (3.5%) | 205 (6.2%) | 134 (4.0%) |  |
| ≥ 3 | 6 (0.7%) | 92 (2.8%) | 43 (1.3%) |  |

SD: standard deviation; IQR: interquartile range; ECMO: extracorporeal membrane oxygenation; PIMS: paediatric multisystem inflammatory syndrome

### **Supplementary Table 6. Results from uni and multivariable regressions for CCU admission – Entire sample**

|  |  | Without CCU N=5497 N (%) | With CCU N=1988 N (%) | univariable | | multivariable | | |
| --- | --- | --- | --- | --- | --- | --- | --- | --- |
|  | Variables |  |  | OR | 95% CI | OR | 95% CI | p value |
| Age |  |  |  |  |  |  |  |  |
|  | 0 to 6 days old | 62 (1.1%) | 68 (3.4%) | 3.10 | [2.19-4.40] | 3.71 | [2.56-5.39] | <.0001 |
|  | 7 days to 2 months old | 1850 (33.7%) | 412 (20.7%) | 1 |  | 1 |  | - |
|  | 3 months to 1 year old | 1164 (21.2%) | 312 (15.7%) | 0.69 | [0.60-0.79] | 0.87 | [0.73-1.03] | 0.1118 |
|  | 2 to 9 years old | 982 (17.9%) | 609 (30.6%) | 2.03 | [1.81-2.28] | 1.19 | [1.00-1.41] | 0.0440 |
|  | 10 to 13 years old | 460 (8.4%) | 255 (12.8%) | 1.61 | [1.37-1.90] | 1.13 | [0.91-1.39] | 0.2652 |
|  | 14 to 17 years old | 979 (17.8%) | 332 (16.7%) | 0.93 | [0.81-1.06] | 0.93 | [0.78-1.11] | 0.4379 |
| Gender | Male | 2910 (52.9%) | 1115 (56.1%) | 1.14 | [1.02-1.26] | 1.05 | [0.94-1.18] | 0.3564 |
| COVID-19 from | |  |  |  |  |  |  |  |
|  | PIMS | 294 (5.4%) | 512 (25.8%) | 6.14 | [5.26-7.17] | 7.17 | [5.97-8.60] | <.0001 |
|  | Respiratory form | 2456 (44.7%) | 838 (42.2%) | 0.95 | [0.86-1.06] | 1.26 | [1.12-1.41] | 0.0001 |
|  | no PIMS no respiratory form | 2747 (50.0%) | 638 (32.1%) | 1 |  | 1 |  | - |
| Number of underlying conditions | |  |  |  |  |  |  |  |
|  | 0 | 4235 (77.04%) | 1145 (57.60%) | 1 |  | 1 |  |  |
|  | 1 | 976 (17.76%) | 621 (31.24%) | 2.35 | [2.09-2.65] | 2.48 | [2.18-2.83] |  |
|  | 2 | 205 (3.73%) | 162 (8.15%) | 2.92 | [2.35-3.63] | 3.38 | [2.69-4.25] |  |
|  | ≥ 3 | 81 (1.47%) | 60 (3.02%) | 2.74 | [1.95-3.85] | 3.19 | [2.24-4.54] | <.0001 |

CCU: critical care unit; OR: Odds ratio; 95% CI: 95% confidence interval; PIMS: paediatric multisystem inflammatory syndrome

### **Supplementary Table 7. Results from uni and multivariable regressions for CCU admission – stratified by age either younger than 2 years old or not**

|  |  |  | without CCU N=3076 N (%) | with CCU N=792 N (%) | univariable | | multivariable | | |
| --- | --- | --- | --- | --- | --- | --- | --- | --- | --- |
|  |  | Variables |  |  | OR | 95% CI | OR | 95% CI | p value |
| < 2 years old | | |  |  |  |  |  |  |  |
|  | Gender | Male | 1702 (55.3%) | 449 (56.7%) | 1.06 | [0.90-1.24] | 1.01 | [0.86-1.19] | 0.9082 |
|  | COVID-19 from | |  |  |  |  |  |  |  |
|  |  | PIMS | 37 (1.2%) | 32 (4.0%) | 3.46 | [2.14-5.59] | 4.05 | [2.46-6.65] | <.0001 |
|  |  | Respiratory form | 1328 (43.2%) | 424 (53.5%) | 1.52 | [1.30-1.78] | 1.49 | [1.26-1.75] | <.0001 |
|  |  | no PIMS no respiratory form | 1711 (55.6%) | 336 (42.4%) | 1 |  | 1 |  | - |
|  | Underlying conditions | |  |  |  |  |  |  |  |
|  |  | Asthma | 65 (2.1%) | 47 (5.9%) | 2.92 | [1.99-4.29] | 2.41 | [1.62-3.60] | <.0001 |
|  |  | Chronic lung disease excluding asthma | 49 (1.6%) | 34 (4.3%) | 2.77 | [1.78-4.32] | 1.26 | [0.76-2.09] | 0.3757 |
|  |  | Sickle-cell disease | 14 (0.5%) | 12 (1.5%) | 3.36 | [1.55-7.30] | 3.75 | [1.70-8.25] | 0.0010 |
|  |  | Cardiovascular disease | 70 (2.3%) | 47 (5.9%) | 2.71 | [1.86-3.95] | 1.79 | [1.17-2.73] | 0.0070 |
|  |  | Neurologic disease | 52 (1.7%) | 28 (3.5%) | 2.13 | [1.34-3.40] | 1.63 | [0.98-2.71] | 0.0578 |
|  |  | Immunocompromised condition | 73 (2.4%) | 38 (4.8%) | 2.07 | [1.39-3.09] | 2.13 | [1.41-3.23] | 0.0004 |
|  |  | Metabolic disease (excluding diabetes) | 34 (1.1%) | 16 (2.0%) | 1.84 | [1.01-3.36] | 0.88 | [0.44-1.72] | 0.7005 |
|  |  | Down syndrome | 8 (0.3%) | 5 (0.6%) | 2.44 | [0.79-7.47] | 1.42 | [0.43-4.76] | 0.5671 |
|  |  | Other comorbidities* | 6 (0.2%) | 5 (0.6%) | 3.24 | [0.99-10.66] | 2.78 | [0.79-9.83] | 0.1129 |
|  |  | Prematurity | 246 (8.0%) | 129 (16.3%) | 2.24 | [1.78-2.82] | 1.89 | [1.47-2.43] | <.0001 |
| ≥ 2 years old | | |  |  |  |  |  |  |  |
|  | Gender | Male | 1208 (49.9%) | 666 (55.7%) | 1.26 | [1.1-1.45] | 1.10 | [0.94-1.28] | 0.2186 |
|  | COVID-19 form | |  |  |  |  |  |  |  |
|  |  | PIMS | 257 (10.6%) | 480 (40.1%) | 5.64 | [4.75-6.71] | 7.01 | [5.73-8.56] | <.0001 |
|  |  | Respiratory form | 1128 (46.6%) | 414 (34.6%) | 0.66 | [0.58-0.76] | 1.02 | [0.86-1.22] | 0.7916 |
|  |  | no PIMS no respiratory form | 1036 (42.8%) | 302 (25.3%) | 1 |  | 1 |  | - |
|  | Underlying conditions | |  |  |  |  |  |  |  |
|  |  | Asthma | 217 (9.0%) | 139 (11.6%) | 1.34 | [1.07-1.67] | 2.10 | [1.64-2.69] | <.0001 |
|  |  | Chronic lung disease excluding asthma | 68 (2.8%) | 52 (4.4%) | 1.57 | [1.09-2.27] | 1.32 | [0.87-1.99] | 0.1895 |
|  |  | Sickle-cell disease | 107 (4.4%) | 78 (6.5%) | 1.51 | [1.12-2.04] | 2.70 | [1.97-3.70] | <.0001 |
|  |  | Obesity | 65 (2.7%) | 42 (3.5%) | 1.32 | [0.89-1.96] | 2.06 | [1.36-3.13] | 0.0007 |
|  |  | Cardiovascular disease | 62 (2.6%) | 96 (8.0%) | 3.32 | [2.39-4.61] | 2.10 | [1.45-3.03] | <.0001 |
|  |  | Neurologic disease | 162 (6.7%) | 119 (10.0%) | 1.54 | [1.20-1.97] | 1.84 | [1.4-2.43] | <.0001 |
|  |  | Immunocompromised condition | 161 (6.7%) | 91 (7.6%) | 1.16 | [0.89-1.51] | 1.21 | [0.90-1.64] | 0.2098 |
|  |  | Diabetes | 66 (2.7%) | 26 (2.2%) | 0.79 | [0.50-1.26] | 1.13 | [0.69-1.86] | 0.6252 |
|  |  | Metabolic disease (excluding diabetes) | 83 (3.4%) | 124 (10.4%) | 3.26 | [2.44-4.34] | 2.97 | [2.15-4.08] | <.0001 |
|  |  | Down syndrome | 19 (0.8%) | 10 (0.8%) | 1.07 | [0.49-2.30] | 0.84 | [0.36-1.97] | 0.6893 |
|  |  | Hepatic and gastric disease | 32 (1.3%) | 13 (1.1%) | 0.82 | [0.43-1.57] | 0.68 | [0.33-1.42] | 0.3066 |
|  |  | Renal disease | 27 (1.1%) | 14 (1.2%) | 1.05 | [0.55-2.01] | 0.95 | [0.46-1.93] | 0.8773 |

The table presents only the underlying conditions with at least 10 patients per modality. *: Other comorbidities: renal disease, hepatic and gastric disease, diabetes

CCU: critical care unit; OR: Odds ratio; 95% CI: 95% confidence interval; PIMS: paediatric multisystem inflammatory syndrome

**Supplementary Figure 1. Number of COVID-19 hospitalizations and of the PIMS (red dotted line) and respiratory (black plain line) forms according to the age and gender of the patient younger than one-year-old**

**
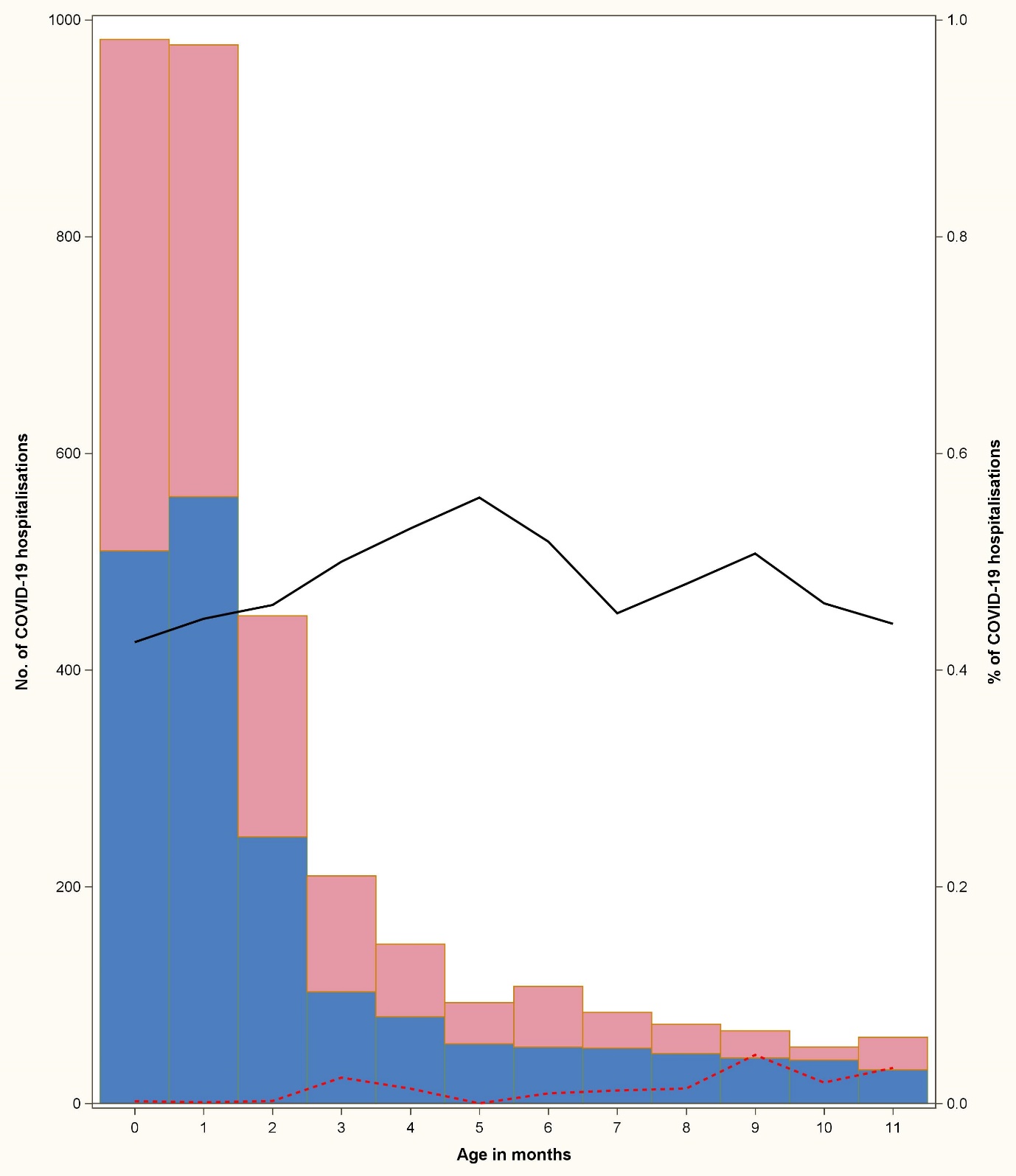
**

The bars give the number of COVID-19 hospitalizations for the males (blue) and the females (pink) separately. The lines give the proportion of the PIMS (red dotted line) and respiratory (black plain line) forms among the hospitalizations.
